# Small Acute Benefits of 4 Weeks Processing Speed Training Games on Processing Speed and Inhibition Performance and Depressive Mood in the Healthy Elderly People: Evidence from a Randomized Control Trial

**DOI:** 10.3389/fnagi.2016.00302

**Published:** 2016-12-23

**Authors:** Rui Nouchi, Toshiki Saito, Haruka Nouchi, Ryuta Kawashima

**Affiliations:** ^1^Creative Interdisciplinary Research Division, Frontier Research Institute for Interdisciplinary Science, Tohoku UniversitySendai, Japan; ^2^Department of Advanced Brain Science, Institute of Development, Aging and Cancer, Tohoku UniversitySendai, Japan; ^3^Human and Social Response Research Division, International Research Institute of Disaster Science, Tohoku UniversitySendai, Japan

**Keywords:** processing speed training, video game, inhibition, depression, cognitive training

## Abstract

**Background:** Processing speed training using a 1-year intervention period improves cognitive functions and emotional states of elderly people. Nevertheless, it remains unclear whether short-term processing speed training such as 4 weeks can benefit elderly people. This study was designed to investigate effects of 4 weeks of processing speed training on cognitive functions and emotional states of elderly people.

**Methods:** We used a single-blinded randomized control trial (RCT). Seventy-two older adults were assigned randomly to two groups: a processing speed training game (PSTG) group and knowledge quiz training game (KQTG) group, an active control group. In PSTG, participants were asked to play PSTG (12 processing speed games) for 15 min, during five sessions per week, for 4 weeks. In the KQTG group, participants were asked to play KQTG (four knowledge quizzes) for 15 min, during five sessions per week, for 4 weeks. We measured several cognitive functions and emotional states before and after the 4 week intervention period.

**Results:** Our results revealed that PSTG improved performances in processing speed and inhibition compared to KQTG, but did not improve performance in reasoning, shifting, short term/working memory, and episodic memory. Moreover, PSTG reduced the depressive mood score as measured by the Profile of Mood State compared to KQTG during the 4 week intervention period, but did not change other emotional measures.

**Discussion:** This RCT first provided scientific evidence related to small acute benefits of 4 week PSTG on processing speed, inhibition, and depressive mood in healthy elderly people. We discuss possible mechanisms for improvements in processing speed and inhibition and reduction of the depressive mood.

**Trial registration:** This trial was registered in The University Hospital Medical Information Network Clinical Trials Registry (UMIN000022250).

## Background

Cognitive function declines with age (Hedden and Gabrieli, 2004). This cognitive decline is one factor of difficulties affecting daily behavior (Burdick et al., 2005; Casutt et al., 2014). Consequently, finding a means of improving cognitive functions is a hot topic in the aging research field (Nouchi and Kawashima, 2014).

Previous studies have revealed that cognitive training, which uses cognitive tasks as training tasks, can improve cognitive functions in the elderly. There are many types of cognitive training such as working memory training (Richmond et al., 2011; Takeuchi et al., 2014), processing speed training (Edwards et al., 2005, 2009a; Wolinsky et al., 2013), memory strategic training (Mahncke et al., 2006; Carretti et al., 2007) and brain training games (Nouchi et al., 2012a). At this moment, a benefit of cognitive training on cognitive function is highly controversial (Lampit et al., 2014; Au et al., 2015; Melby-Lervag et al., 2016; Simons et al., 2016). For brain training games, an online cognitive training game improved cognitive functions in the older adults (Corbett et al., 2015), but not in the young adults (Owen et al., 2010). On the other hand, a brain training game such as Brain Age improved cognitive functions in both young and older adults (Nouchi et al., 2012a, 2013). In addition, meta-analysis studies for working memory training reported different conclusions (Au et al., 2015, 2016; Melby-Lervag and Hulme, 2016; Melby-Lervag et al., 2016).

However, one meta-analysis study, which comparted the effects of different types of cognitive training for older adults, revealed that processing speed training has stronger effects on cognitive function compared to other cognitive training (Papp et al., 2009). Indeed, earlier studies have demonstrated that processing speed training provides better cognitive benefits than memory or reasoning training. Moreover, the benefits of processing speed training remained several years later (Jobe et al., 2001; Ball et al., 2002; Willis et al., 2006). In addition, processing speed training had positive effects on emotional aspects such as depressive symptoms (Wolinsky et al., 2009) and health-related quality of life (Wolinsky et al., 2006). Consequently, processing speed training is an effective intervention program in terms of its cognitive and emotional benefits.

Although previous studies have demonstrated beneficial effects of processing speed training on cognitive functions (Jobe et al., 2001; Ball et al., 2002; Willis et al., 2006; Wolinsky et al., 2013) and mental health (Wolinsky et al., 2006, 2009), some unresolved issues persist (training task, intervention period, comparison group). First, previous processing speed training (Jobe et al., 2001; Ball et al., 2002; Willis et al., 2006; Wolinsky et al., 2013) was based on the useful field of view (UFOV), which is a cognitive assessment for visual processing speed (Ball et al., 1988). Additionally, processing speed training has traditionally been conducted using a desk top PC with trainers at laboratories (Ball et al., 2002) or at home (Wolinsky et al., 2013). To generalize the effects of processing speed training, it is an important to question whether or not other types of processing speed training can improve cognitive functions and emotional states. Second, because previous studies using processing speed training examined the effects of long-term intervention periods (1 year) (Ball et al., 2002; Wolinsky et al., 2013), it remains unclear whether or not processing speed training can improve cognitive functions and emotional states during short-term training periods, or not. Considering the intervention cost, it is an important to test positive effects of processing speed training using a short-term period. Checking benefits of short-term processing speed training would also help to generalize the beneficial effects of processing speed training. Third, numerous previous studies have used a no intervention group as a comparison group (Ball et al., 2002). Such a study design cannot exclude placebo effects, as reports have pointed out (Wolinsky et al., 2013). For assessing the effects of processing speed training on emotional states (Wolinsky et al., 2006, 2009), no study has examined active control groups as a comparison group. To accumulate clear evidence of processing speed training on cognitive function and emotional states, it is necessary to use an active control group as a comparison group.

This study was designed to investigate these unresolved issues. For unresolved issues related to training tasks, we newly developed video-game-like processing speed training games that can be used on a tablet PC. All training games require processing speed (please see Methods). Additionally, to test whether processing speed training using short-term intervention period has beneficial effects, we set a 4 week intervention period because previous intervention studies have shown improvements of cognitive functions after 4 week intervention periods (Nouchi et al., 2012a, 2014). Finally, we used an active control group that uses the same training period and a similar training setting. To control the effects of new experiences such as doing some cognitive task (or playing video games), using a new device, and social interaction, we also developed a knowledge quiz training game that can be played on a tablet PC. The knowledge quiz training game simply asked participants to respond with knowledge without time pressure. The processing speed training and knowledge quiz training games were conducted at the homes of subjects using the same tablet PC. By using this active control group with a knowledge quiz training game, one can reduce the shortcomings of earlier studies that are described above.

This study was designed to investigate the beneficial effects of the processing speed training game on the cognitive function and emotional state of healthy elderly people. For this study, we conducted a 4 week single-blinded randomized controlled trial (RCT) with two parallel groups: the processing speed training game (PSTG) group and the active control group using the knowledge quiz training game (KQTG). Participants were blind to the study’s hypothesis and the group membership of participants. To clarify the benefits of the new developed processing speed training game, we assessed a broad range of cognitive functions (inhibition and shifting performance in executive function, episodic memory, short-term memory, working memory, reasoning, and processing speed) and several emotional states (quality of life and mood states). Based on previous findings (Ball et al., 2002; Wolinsky et al., 2006, 2013), we expected that the new developed processing speed training game would lead to greater improvement of cognitive function and emotional state compared to that experienced by the active control group.

## Method

### Randomized Controlled Trial Design and Setting of This Trial

This RCT was conducted in Sendai city, Japan. The study protocol of this study was approved by the Ethics Committee of the Tohoku University Graduate School of Medicine. This RCT was registered in the University Hospital Medical Information Network (UMIN) Clinical Trial Registry (UMIN000022250).

**FIGURE 1:**
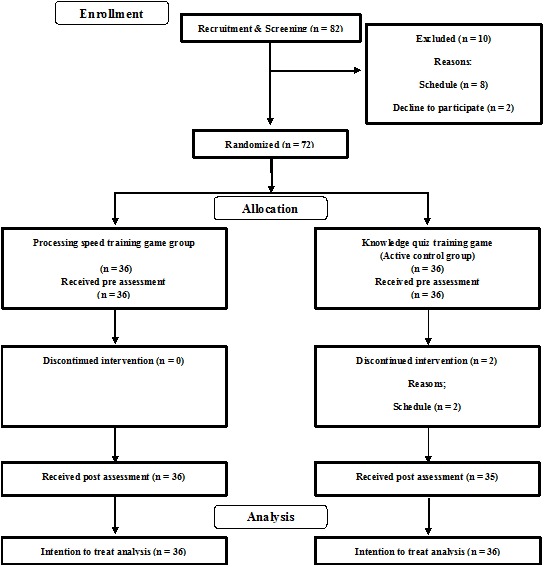
**Consolidated standards of reporting trial (CONSORT) diagram**.

To investigate the benefits of processing speed training games on cognitive function and emotional state in healthy older adults, we conducted a single-blinded RCT with the active control group. All participants were blinded to the study’s hypothesis and the group membership of participants. The Consolidated Standards of Reporting Trials (CONSORT) statement^1^ (see Supplementary Table 1) was used to report the study structure. The RCT design is presented in **Figure 1**.

### Participants

Eventually, 72 older people participated in this study. First, 82 participants were recruited through the local newspaper (Kahoku Weekly) (**Figure 1**). Then, 82 interested participants contacted the research group by telephone. At this time, they were screened using a semi-structured telephone interview. RN and HN conducted the semi-structured interview. They had more than 5 years’ experiences in the aging research field. The interview took about 5 min. We basically asked participants 10 questions related to our inclusion and exclusion criteria [(1) age, (2) sex, (3) experience for intervention studies, (4) mother tongue, (5) handedness, (6) subjective memory function, (7) history of medication and disease, (8) blood pressure, (9) diabetes, and (10) training schedule]. We used the semi-structured interview because an order of questions and details of question was flexible (e.g., details of history of medication is changed based on the participants answers). From results of the screening, eight people were excluded because of their schedule; two declined to participate because of health problems. We received written informed consent from all remaining participants [*n* = 72, number of men = 28, number of female = 44, average age = 68.92 (*SD* = 3.70)], then they were invited to visit Tohoku University for more detailed cognitive functional screening assessment using the Mini-Mental State Examination (MMSE) (Folstein et al., 1975). No participant was excluded based on the MMSE score. **Table 1** presents the baseline characteristics. Ratios of people who have a tablet PC (25% in PSTG, 22% in KQTG) and who play video games on a PC or smart phone (31% in PSTG, 28% in KQTG) were almost equal in both groups. No significant differences were found between the groups in any data (two-sample *t*-test, *p* > 0.10). The score of MMSE was within the normal range.

**Table 1:** Age, education years, cognitive function score, and mental health score in both groups at baseline.

	PSTG group		KQTG group	
	Mean	*SD*	Mean	*SD*	*p*-value
Age (year)	69.14	3.70	68.88	3.73	0.77
Education (year)	12.44	3.49	11.82	3.43	0.46
MMSE (score)	28.36	1.59	28.5	1.29	0.69
Raven (number)	34.00	2.74	33.79	3.48	0.78
LM (score)	8.56	3.56	9.35	3.55	0.34
Cd (number)	73.89	12.62	73.38	14.01	0.87
SS (number)	35.08	7.38	35.68	5.55	0.70
DS-F (score)	5.94	0.95	5.82	0.94	0.59
DS-B (score)	4.53	1.48	4.15	0.99	0.20
LFT (number)	9.14	2.55	9.71	2.64	0.36
CFT (number)	12.39	2.45	12.41	2.56	0.97
rST (number)	47.64	9.63	45.94	8.33	0.43
ST (number)	33.00	8.85	31.24	6.22	0.33
Mental health in SUBI (score)	39.22	6.36	38.11	6.74	0.47
Mental fatigue in SUBI (score)	52.50	6.56	53.47	5.29	0.49
T-A in POMS (score)	6.47	2.29	6.86	2.21	0.47
D in POMS (score)	6.69	2.41	6.08	2.35	0.28
A-H in POMS (score)	6.11	2.15	6.17	1.89	0.91
V in POMS (score)	6.69	2.44	7.36	2.28	0.23
F_I in POMS (score)	4.22	2.23	4.72	2.28	0.35
C in POMS (score)	2.00	1.91	2.69	2.08	0.14
F in POMS (score)	5.50	1.80	5.97	1.84	0.27

*Group comparison (two sample *t*-tests) of the pre-training scores revealed no significant difference for any measures (*p* > 0.10). PSTG, processing speed training game; KQTG, knowledge quiz training game; MMSE, mini mental state examination; Raven, raven’s colored progressive matrix test; LM, logical memory; Cd, digit symbol coding; SS, symbol search; DS-F, digit span forward; DS-B, digit span backward; LFT, letter fluency task; CFT, category fluency task; rST, reverse stroop test; ST, stroop test; SUBI, subjective well-being inventory; POMS, profile of mood state; T-A, tension-anxiety; D, depression-dejection; A-H, anger-hostility; V, vigor activity; F-I, Fatigue-Inertia; C, confusion-bewilderment; F, friendliness; SD, standard deviation.*

### Inclusion and Exclusion Criteria

This study was conducted to investigate benefits of processing speed training game on cognitive function and emotional state in healthy elderly people. Based on our previous studies, we used the following inclusion and exclusion criteria. The following descriptions in this section are mostly reproduced from our earlier report (Nouchi et al., 2012b). “The criteria include participants who report themselves to be right-handed, native Japanese speakers, unconcerned about their own memory functions, not using medications known to interfere with cognitive functions (including benzodiazepines, antidepressants or other central nervous agents), and having no disease known to affect the central nervous system, including thyroid disease, multiple sclerosis, Parkinson disease, stroke, severe hypertension (systolic blood pressure is over 180, diastolic blood pressure is over 110), and diabetes. Age of participants is over 60 years old” (Nouchi et al., 2012b). Criteria exclude participants who have an MMSE score of less than 26. Participants participating in another cognition-related intervention studies were excluded.

### Sample Size

We calculated the sample size using G^∗^ power (Faul et al., 2009). The sample size was based on the changed score of symbol search coding. We expected that we detected medium effect size values (η^2^ = 0.12) because a previous intervention study (Nouchi et al., 2012a) using brain training games in the healthy elderly showed a medium effect size in SS (η^2^= 0.12). To calculate the samples size, we set an analysis of covariance (ANCOVA) model using a pre-intervention score of processing speed, sex, and age as covariates with a two tail test, α = 0.05, and 0.85 power. The sample size was calculated with a 5% drop out ratio, indicating 36 participants per group (total 72 participants).

### Randomization

After receiving the signed informed consent forms from participants, participants were assigned randomly to the processing speed training game group or to an active control group using a random draw program^2^. We stratified participants based on sex and used a blocked randomization (block size; 2) with an allocation ratio of 1:1.

### Overview of Intervention

Participants were asked to play PSTG or KQTG at home for 15 min, at least 5 days per week during 4 weeks. The intervention schedule was set based on that used in an earlier study (Nouchi et al., 2012a). Both training games were used on a tablet PC. Consequently, all participants played the training game using a touch panel screen. Training game scores, training time, and training day were automatically recorded in the tablet PC. Thus, we can check whether or not participants conducted training game based on the planned methodology. Participants cannot play the training game for more than 15 min per day because our training programs automatically were terminated after 15 min. At the end of each training day, participants reported their subjective feelings of satisfaction and enjoyment when playing the game immediately after training. We administered cognitive measure tests and checked emotional states 1 day before and after the 4-week intervention period. On the first day of the intervention period, participants were provided a tablet PC on which had already been installed either of PSTG or KQTG. For 1 h, they received instructions on how to use the tablet PC and how to play the training game. After finishing each training game, training game performance was automatically recorded on the tablet PC. Finally, the tablet PCs were returned on the final day.

### Processing Speed Training Game Group

**FIGURE 2:**
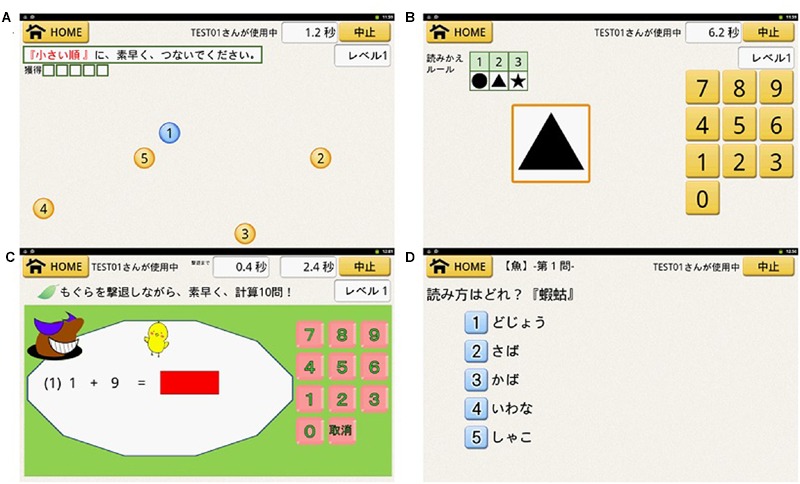
**A processing speed training game and knowledge quiz training games.** For connection numbers **(A)**, participants connect numbers in number order. In the case of **(A)**, participants are asked to draw the lines from a smaller number to a big number as quick as possible (1→2→3→4→5). For translating figures to numbers **(B)**, participants select numbers related to target figures. The rule is presented at the top left corner of the screen. The numeric keypad (response pad) is present at right side of the screen. In the case of **(B)**, circle correspond to 1, triangle correspond to 2, and star correspond to 3. The rule is changed every game. The target figure is presented around the center of the screen. In the case, participants are asked to touch the specific number (3) related to the target figure (triangle) as quick as possible. For calculation with whack-a-mole **(C)**, participants conduct simple calculations with tapping targets. The numeric keypad (response pad) is present at right side of the screen. In the case of **(C)**, the tapping target is a mole. A numerical expression is presented around the center of the screen (e.g., 1 + 9). The tapping target is moving until participant tap the target and the number of the tapping target increase during the game. In the case, participants are asked to response the answer of the numerical expression (10) and to tap the target as quick as possible. For the meaning of words **(D)**, participants answer a pronunciation of Kanji. A quiz is presented at the top of the screen. In the case of **(D)**, the target Kanji (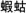: squilla) is presented. The five pronunciations are presented. In the case, the first pronunciation is dojyou (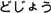: loach), the second pronunciation is saba (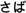: mackerel), the third pronunciation is kaba (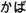: hippopotamus), the fourth pronunciation is iwana (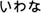: char), the fifth pronunciation is syako (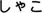: squilla). Participants are asked to select the corrected pronunciation of the target Kanji (in this case, the corrected answer is the fifth pronunciation: syako).

We developed 12 processing speed training games to function on the tablet PC (**Figure 2**). The games concepts were based on earlier processing speed training studies (Ball et al., 2007). In processing speed training, all training games include localization, detection, or identification elements. These processing training games were developed by pilot studies. All participants in the pilot studies were healthy elderly and did not participated in the RCT. In the first pilot study (*N* = 57), we checked the task difficulties in each game using game performance (reaction time). Based on results of the first pilot study, we set task difficulty (game level). In the second pilot study (*N* = 47), participants were asked to play PSTG for 4 weeks. We confirmed that participants did the PSTG for 4 weeks and the game level in each training game was increased through the intervention period. Finally, we checked the correlations (*N* = 18) between the performance of processing speed task (Cd and SS) and the performance of processing speed training game. We confirmed that performances of training games (reaction time) were correlated to performances of SS and Cd (*r* = from 0.4 to 0.7). People with higher processing speed abilities showed higher game performance. These results suggested the validity of processing speed training games.

For connection numbers, participants were asked to connect numbers in number order. For reverse connection numbers, participants were asked to connect numbers in reverse number order. For translating figures to number, participants were asked to select numbers related to figures. For calculation with whack-a-mole, participants were asked to conduct simple calculations with tapping targets. For lining up numbers, participants were asked to push numbers in number order. For reverse lining up numbers, participants were asked to push numbers in reverse number order. For counting balls, participants were asked to count the balls. For a ball-toss game, participants were asked to toss the colored ball into the same colored cage. For copying forms, participants were asked to copy the same forms in panels based on a target. For hide and seek, letter (number) sequences were presented in which one letter (number) was hidden (e.g., 12_45). Participants were asked to select the correct number or letter that matched the sequence (e.g., 3). For finding shapes, participants were asked to find the corrected shapes based on the instructions. For finding letters, participants were asked to select the correct answer based on instructions (e.g., transform Chinese characters that express numbers to number or simple calculations). All games required participants to detect, identify, discriminate, and localize targets as quickly as possible. After finishing each training game, training game performance was automatically recorded on the tablet PC. Thus, we can check whether or not participants conducted training game based on the planned methodology. To keep motivation for playing the game and to increase effects of PSTG, we set the following rules. First, a participant can play only three games at the first day, we added new games daily (finally total 12 games). Second, the difficulty (level) of each game was changed from level 1 to level 8 based on participants’ game performance. Consequently, this PSTG is intensive and adaptive training. Participants were asked to play the training games equally. Additionally, the training programs cannot allow participants to play the same training three times in a row. Thus, each training game took approximately the same amount of time on the each training day (15 min).

### Knowledge Quiz Training Game Group (Active Control Group)

We developed five knowledge quiz training games for use on the tablet PC (**Figure 2**). Meaning of words is a quiz that includes antonyms and synonyms. Reading of words is a quiz for pronunciation of kanji. Kanji idioms are a quiz that asks players about a four character-idiom. The Japanese literature quiz asks players about Japanese authors of novels and haiku (Japanese 17 mora poems). Quizzes for Japanese society ask players about Japanese geography, castles, and cities. To reduce elements of processing speed in KQTG, we set a fixed time for answers (10 s). The quiz did not proceed if participants answered briefly. We also required participants to think about correct answers without rushing. After finishing each game, game performance was automatically recorded on the tablet PC. All quizzes used information selected from Japanese dictionaries. To maintain motivation for playing games, we added new stages to each game based on participants’ performance. All games had eight stages with similar levels of difficulty. Adding new stages simply increases the number of quizzes in each game. Consequently, KQTG was not intensive adaptive training. Participants were asked to play the training games equally. Additionally, the training programs cannot allow playing the same training three times in a row. Thus, each training game took approximately the same amount of time on the each training day.

### Cognitive Functional Measures

To check the benefits of the processing speed training game on cognitive functions, we conducted the following cognitive functional measures such as reasoning ability, processing speed, executive function, short term/working memory, and episodic memory). We also check a cognitive status using MMSE. It took about 2 h to conduct all cognitive tests. For checking a cognitive status briefly, we used MMSE, which measure memory, attention, language, and visuospatial abilities (Folstein et al., 1975). For reasoning ability, we used Raven’s Colored Progressive Matrices Test (Raven) (Raven, 1965; Smits et al., 1997). In this test, participants were asked to select the correct figure which matched the target figure. For processing speed, we used digit symbol coding (Cd) and symbol search (SS) from WAIS-III (Wechsler, 1997). The following descriptions in Cd and SS are mostly reproduced from our earlier report (Nouchi et al., 2012b). “For Cd, the participants were shown a series of symbols that were paired with numbers. Using a key within a 120 s time limit, participants draw each symbol under its corresponding number. The primary measure of this test was the number of correct answers. In SS, participants visually scanned two groups of symbols (a target group and a search group) and reported whether either of the target symbols matches any symbol in the search group. Participants responded to as many items as possible within a 120 s time limit. The primary measure of this test was the number of correct answers.” To measure shifting ability in executive function, we used Japanese version’s letter fluency task (LFT) and category fluency task (CFT) (Ito et al., 2004). The following descriptions in LFT and CFT are mostly reproduced from our earlier report (Nouchi et al., 2012b). “In LFT, a Japanese letter, ‘ka,’ was given to each participant, who was then asked to generate common nouns beginning with this letter—as many as possible in 60 s. In CFT, a category name (Animal) was given to each participant, who was then asked to generate many words of a certain category (Animal). The participants were instructed not to include proper nouns or to repeat one that had already been stated.” To measure inhibition ability in executive function, we used Stroop task (ST) and reverse Stroop task (rST) (Hakoda and Sasaki, 1990; Watanabe et al., 2011). “In the ST, in the leftmost of six columns, a word naming a color was printed in another color (e.g., ‘red’ is printed in blue letters) and the other five columns contain words naming colors. Participants must check the column containing the word naming the color of the word in the leftmost column. In the reverse ST, in the leftmost of six columns, a word naming a color was printed in another color (e.g., ‘red’ is printed in blue letters); the other five columns were filled respectively with five different colors from which participants must check the column with the color matching the written word in the leftmost column. In each task, participants were instructed to complete as many of these exercises as possible in 1 min. The primary measure for this task is the number of correct items” (Nouchi et al., 2012b). To measure short-term memory, we used digit span forward (DS-F). To measure working memory, we used digit span backward (DS-B). DS-F and DS-B are subtests of WAIS III (Wechsler, 1997). The following descriptions in DS-F and DS-B are mostly reproduced from our earlier report (Nouchi et al., 2012b). “For DS-F, participants repeated numbers in the same order as they were read aloud by the examiner. For DS-B, participants repeated numbers in the reverse order of that presented aloud by the examiner. In both, the examiner read a series of number sequences which the examinee must repeat in either forward or reverse order. DS-F has 16 sequences. DS-B had 14 sequences. The primary measures of this test were raw scores that reflect the number of correctly repeated sequences until the discontinue criterion (i.e., failure to reproduce two sequences of equal length) was met. The maximum raw score of DS-F was 16. The maximum raw score of DS-B was 14.” To measure episodic memory, we used logical memory (LM) from WMS-R (Wechsler, 1987). In LM, “LM consisted of two short-paragraph-length stories (Story A and Story B). In LM, participants must memorize the short story. The stories were scored in terms of the number of story units recalled, as specified in the WMS-R scoring protocol. We used either Story A or Story B. The primary measure for this task was the number of correct story units recalled” (Nouchi et al., 2012b).

### Emotional State Measure

To investigate the beneficial effects of PSTG on emotional states and quality of life, we used a short version Profile of Mood State second edition (POMS2) (Heuchert and McNair, 2012; Yokoyama and Watanabe, 2015) and World Health Organization Subjective Well-being Inventory (WHO-SUBI) (Sell, 1994; Ono and Yoshimura, 2001). POMS2 has total 35 items with 5 point scales. These items are divided into 7 subscales: Tension-Anxiety (T-A), Depression-Dejection (D), Anger-Hostility (A-H), Vigor-Activity (V), Fatigue-Inertia (F-I), Confusion-Bewilderment (C), and Friendliness (F). WHO-SUBI has 40 questions using 3 point scales. WHO-SUBI has 2 subdomains for quality of life such as mental health and mental fatigue. POMS2 and WHO-SUBI were standardized in Japanese population. All measures have high reliability and high validity (Ono and Yoshimura, 2001; Yokoyama and Watanabe, 2015).

### Analysis

First, we calculated the standardized pre scores and the standardized changed score (post-intervention score minus pre-intervention score) in cognitive function and emotional states using the following standardized norm (*Z*-score, Mean = 100, *SD* = 10). The reason of using standardized scores is to meet the assumptions of *F*-tests such as normality or homogeneity. Second, we conducted one-tailed ANCOVA (analysis of covariance) for the all standardized changed scores because we had strong hypothesis that PSTG would have more positive effects compared to KQTG. The standardized changed scores was the dependent variable. Group was the independent variable. The standardized pre-score in the dependent variable, age, and sex were used as covariates. Finally, we used false discovery rate (FDR) correction methods to adjust all *p* values (Benjamini et al., 2006). All participants were included based on the intention to treat principle. Missing data were imputed using the expectation-maximization method which imputes missing values using maximum likelihood estimation with observed data in an iterative process. All analyses were conducted using SPSS ver. 18.

## Results

Two participants in KQTG were dropout because of schedules during the intervention period. Based on an intention to treat rule, we imputed missing values of 2 participants in KQTG. No significant difference was found in the average number of training days between PSTG (*M* = 27.06, *SD* = 1.71) and KQTG (*M* = 26.68, *SD* = 2.46). Levels of each game in PSTG and stages of each game in KQTG are presented in Supplementary Tables 2 and 3. We averaged the satisfaction and enjoyment scores during playing game each week. No significant difference was found in the average scores in the satisfaction and enjoyment between groups (**Table 2**). There were no significant differences of all measures at baseline (**Table 1**). All raw scores after the intervention period are shown in Supplementary Table 4.

**Table 2:** Average scores of satisfaction and enjoyment in both group every week.

	Processing speed game training game		Knowledge quiz game training group	
	Mean	*SD*	Mean	*SD*	*p*-value
**Satisfaction**
1 week	5.38	1.73	5.23	2.18	0.75
2 weeks	5.16	1.73	5.37	2.04	0.65
3 weeks	4.98	1.58	5.34	2.10	0.41
4 weeks	4.98	2.01	4.72	2.38	0.62
**Enjoyment**
1 week	6.58	1.61	6.21	2.23	0.43
2 weeks	6.13	1.66	6.09	2.18	0.94
3 weeks	5.82	1.59	5.91	2.17	0.83
4 weeks	5.66	2.01	5.19	2.51	0.39

*No significant difference was found between groups (two-sample *t*-test, *p* > 0.10). SD, standard deviation.*

To check the benefit of PSTG on cognitive functions and emotional state, we conducted ANCOVAs for standardized changed scores (**Table 3**). For cognitive functions, we found significant improvements in two cognitive domains (processing speed and inhibition). For processing speed, PSTG showed significant improvement in the Cd score [*F*(1,67) = 6.05, η^2^ = 0.08, *adjusted p* = 0.03] and the SS score [*F*(1,67) = 6.63, η^2^ = 0.08, *adjusted p* = 0.03]. For inhibition, PSTG showed significant improvement in the rST score [*F*(1,67) = 6.61, η^2^ = 0.09, *adjusted p* = 0.03] and the ST score [*F*(1,67) = 7.41, η^2^ = 0.09, *adjusted p* = 0.03]. For the mental health state, PSTG showed improvements of depression score in POMS [*F*(1,67) = 6.09, η^2^ = 0.05, *adjusted p* = 0.03]. It is important to note that the effects sizes (η^2^) of Cd, SS, rST, ST, and depression score were small (from = 0.05 to 0.09).

**Table 3:** Standardized changed score in cognitive function score, and mental health score in both groups.

	PSTG group		KQTG group			
	Mean	*SD*	Mean	*SD*	*adjusted p*-value	Effect size (η^2^)
MMSE (score)	101.41	10.47	98.59	9.44	0.17	0.00
Raven (number)	99.30	7.72	100.70	11.93	0.38	0.00
LM (score)	100.34	9.37	99.66	10.72	0.40	0.08
Cd (number)	102.63	10.46	97.37	8.91	0.03	0.08
SS (number)	102.87	9.38	97.13	9.90	0.03	0.01
DS-F (score)	100.74	10.68	99.26	9.37	0.32	0.02
DS-B (score)	100.66	11.53	99.34	8.30	0.17	0.00
LFT (number)	100.24	10.50	99.76	9.62	0.40	0.00
CFT (number)	100.45	7.95	99.55	11.80	0.39	0.00
rST (number)	102.65	8.75	97.35	10.57	0.03	0.09
ST (number)	102.58	8.50	97.42	10.82	0.03	0.09
Mental health in SUBI (score)	99.31	9.38	100.69	10.67	0.39	0.00
Mental fatigue in SUBI (score)	101.48	8.31	98.52	11.37	0.29	0.01
T-A in POMS (score)	100.99	10.86	99.01	9.11	0.38	0.00
D in POMS (score)	96.95	11.85	103.05	6.57	0.03	0.05
A-H in POMS (score)	99.60	10.40	100.40	9.71	0.39	0.00
V in POMS (score)	100.37	10.19	99.63	9.94	0.40	0.00
F_I in POMS (score)	101.06	11.45	98.94	8.34	0.39	0.00
C in POMS (score)	99.69	10.68	100.31	9.41	0.29	0.01
F in POMS (score)	101.58	10.73	98.42	9.09	0.29	0.01

*PSTG, processing speed training game; KQTG, knowledge quiz training game; MMSE, mini mental state examination; Raven, raven’s colored progressive matrix test; LM, logical memory; Cd, digit symbol coding; SS, symbol search; DS-F, digit span forward; DS-B, digit span backward; LFT, letter fluency task; CFT, category fluency task; rST, reverse stroop test; ST, stroop test; SUBI, subjective well-being inventory; POMS, profile of mood state; T-A, tension-anxiety; D, depression-dejection; A-H, anger-hostility; V, vigor activity; F-I, Fatigue-Inertia; C, confusion-bewilderment; F, friendliness; SD, standard deviation.*

## Discussion

For this study, we developed the new tablet PC version of PSTG. We then investigated the benefits of PSTG for cognitive functions and emotional states of healthy elderly people. Results support two main findings. First, our results showed that the 4-week PSTG intervention improved cognitive performance in processing speed measured by Cd and SS and inhibition of executive function performance measured by ST and rST compared to the active control group. Second, PSTG demonstrated the improvement of depressive mood, as measured by POMS. Taken together, results of this study extend previous evidence by demonstrating improvements of cognitive functions and emotional states using a 4-week intervention period. Each finding is discussed in separate sections below. It should be noted that the two main findings were based on only the acute benefits of PSTG and the effects sizes of the results were small. In addition, we did not conduct a follow up measure, it is not clear whether new PSTG has a long-term benefits. These points are also discussed in limitations

The first main finding is that a 4-week period of processing speed training can improve processing speed and inhibition of executive functions. For processing speed, previous reports of some studies have described that 1 year processing speed training improved the processing speed performance measured by UFOV and Cd (Ball et al., 2002, 2007; Wolinsky et al., 2013). Supporting previous findings, this study replicated the improvement of Cd and demonstrated the improvement of SS after processing speed training. These results suggest that processing speed training in both short-term and long-term intervention periods has positive effects on processing speed performance. On the other hand, this study did not use more simple and basic processing speed tasks such as inspection time and hick reaction time tests. It is unclear that the short-term processing speed training has benefits of these simple and basic processing speeds. Previous study demonstrated that performance of Cd and SS are affected by other cognitive process such as memory (Piccinin and Rabbitt, 1999). Inspection time can measure more pure mental speed and reflect cognitive aging (Ritchie et al., 2014). To generalize the current results, in the future study we should use the simple and basis processing speed tasks.

For executive function, one earlier study showed that processing speed training improved the shifting performance in executive functions measured by a trail making test (Wolinsky et al., 2013). We did not replicate the improvement of shifting performance in executive function measured by LFT and CFT, but we first demonstrated the improvement of inhibition performance in executive function measured by ST and rST. Some methodological differences exist A first point is an intervention period. We used a 4-week intervention period, but an earlier study used a 1-year intervention period (Wolinsky et al., 2013). The possibility exists that a 4-week intervention period would be insufficient to facilitate a change of performance. A second point is the difference of cognitive functional measures. Previous studies used the trail making test as a shifting performance measure. The trail making test requires participants to draw a line between numbers and letters (Reitan, 1955). It depends more on visual ability. By contrast, we used LFT and CFT as a shifting performance measure. LFT and CFT are a part of a verbal fluency task that depends on the shifting performance in a verbal domain (Ito et al., 2004). Many training tasks in PSTG were non-verbal training tasks. Therefore, we did not find significant improvement of the verbal shifting performance. To investigate the improvements of shifting performance after processing speed training, further studies using long-term intervention period and several types of cognitive functional measures in one cognitive domain are necessary.

The improvements of processing speed and inhibition are explained by the overlapping hypothesis (Nouchi et al., 2013, 2016; Nouchi and Kawashima, 2014). The overlapping hypothesis assumes that cognitive functions would be improved by cognitive training when training tasks and untrained cognitive functional measures share common mental processes. In this study, participants were asked to do several processing speed training tasks during 4 weeks. The processing speed training tasks involved processing speed and inhibition processes. For example, processing speed is the most important factor to conduct training tasks as quickly as possible. Inhibition processes also play an important role in selecting targets, ignoring distractors, and using appropriate rules in each training game. Based on the overlapping hypothesis, the improvements of processing speed and inhibition performance are explainable by the following mechanism. The processing speed training games requires the mental processes mentioned above. The processing speed training and cognitive functional measures share similar mental process. During conduct of the processing speed training game, mental processes related to processing speed and inhibition would be used. They are expected to be facilitated. Therefore, processing speed and inhibition were improved after the processing speed training game.

The Cattel–Horn–Carroll (CHC) model (Schneider and McGrew, 2012) would support the idea of overlapping hypothesis. The CHC presents three levels of cognitive process: narrow abilities, broad abilities, and general ability. For example, the broad ability of processing speed consists of several narrow abilities such as perceptual speed, rate of test-taking, number facility, reading speed, and writing speed. Interestingly, one recent paper using a confirmatory factor analysis (Jewsbury et al., 2016) revealed that the narrow ability of inhibition was included the broad ability of processing speed factor. In addition, the narrow ability of switching is separable from the broad ability of processing speed factor. Based on these facts, results of this study may be explained by the following idea. PSTG would require the broad ability of processing speed which consists of narrow abilities such as perceptual, reading, motor speed and inhibition. Therefore, PSTG improved performance of the included narrow abilities measured by Cd, SS, ST, and rST. Because PSTG did not include other narrow abilities such as memory, shifting, and reasoning, PSTG did not improve the performance of other narrow abilities.

The second main finding is reduction of the depressive mood in POMS after 4 weeks of processing speed training. An earlier study demonstrated that long-term processing speed training for healthy older adults led to reduction of depressive symptoms, as measured by Center for Epidemiologic Studies Depression Scale (CES-D) (Wolinsky et al., 2009). One cognitive training study for healthy young adults showed that 4 weeks of working memory training reduced the depressive mood measures by POMS (Takeuchi et al., 2014). This study extends the previous evidence by demonstrating the reduction of depressive mood in healthy older adults after 4 weeks’ processing speed training. Previous studies reported the relationship between processing speed performance and depressive mood in younger patients (Tsourtos et al., 2002) and normal older adults (Baune et al., 2006). It is still unclear the causal relationship between processing speed and depressive mood because previous studies were cross-sectional studies. Our intervention study suggests a possibility that the change of processing speed performance would lead a change of depressive mood in healthy older adults. However, this study showed the only acute benefits of depressive mood after 4 weeks intervention period, it would be needed to investigate the long-term effect of processing speed training using a follow up phase on depressive mood.

It is important to consider the meaning of the depressive mood change measures by POMS. The current results showed the only benefit of processing speed training on depressive mood in the healthy older sample. Previous study (Gibson, 1997) showed that the depressive mood measured by POMS was highly significantly correlated with depressive symptom scale measured geriatric depression scale (Yesavage et al., 1982). However, the change of the depressive mood measured by POMS did not directly mean a relief of depressive symptoms because participants in this study were healthy older adults. To specify the benefit of processing speed training on depressive symptom, it should be needed to conduct a RCT for depression patients.

Two potential mechanisms can be inferred for reduction of a depressive mood after processing speed training. The first potential mechanism is that processing speed training functions as emotional regulation. One earlier study demonstrated that cognitive tasks reduced negative emotions (Iida et al., 2011, 2012). Conducting processing speed training games would present one distraction that might induce a person to ignore negative experience tasks or divert attention away from emotional experiences. Therefore, depressive emotion declined after processing speed intervention. The second potential mechanism is that processing speed training directly affects brain function and structures related to negative mood. A recent report described that cognitive intervention using working memory training reduced negative emotions such as depression and anxiety and led to change brain activity in the insula (Takeuchi et al., 2014). The insula is associated with feeling of negative emotion (Kober et al., 2008) and depressive symptoms (Lagopoulos et al., 2012; Whalley et al., 2013). As shown also by an earlier study (Takeuchi et al., 2014), the possibility exists that processing speed training engenders changes in brain functions related to negative emotion states and control of emotion. The reduction of depressive mood might be explained by these neural changes. However, these brain mechanisms related to change of depressive mood is a quite speculative. In future studies, it should be important to investigate these potential mechanisms to improve emotional states using neuroimaging studies or assessments for emotional regulation experience.

To familiarize the general public with cognitive training, it is important to reduce costs for intervention and to develop user-friendly intervention tools. Results of this study can contribute to these issues. First, compared to previous findings, this study clarified some benefits of 4 weeks of processing speed training: it can improve the cognitive function and emotional state of healthy elderly people. Shortening the intervention periods can be expected to reduce the intervention costs (time and money). Second, we developed the processing speed training game to function on a tablet PC and presented scientific evidence. Cognitive training using a tablet PC can be an effective and easy means of doing cognitive training anytime for elderly people. Therefore, we believe that our study can provide new, useful, and effective tools for cognitive training.

The sample size of the RCT was estimated based on about 5% dropout ratio. However, the current dropout ratio was about 15% (12 participants dropped out in this study). The main reason of the dropout (10 participants) was schedule (e.g., some events such as important family events or social events came up unexpected or they cannot coordinate the schedule for cognitive functional tests). In addition, eight participants dropped out before interventions. This reason was not directly related to the intervention program. Thus, the greater dropout ratio would not affect the current result.

This study has some limitations. This study was conducted to test whether short-term processing speed training can improve the cognitive functions and emotional states of healthy elderly people. It remains unclear how long the benefits of short-term processing speed last. Previous studies have found that the benefits of 1-year processing speed training persisted for 5 years (Willis et al., 2006). Investigating the long-term benefits of the short-term processing speed training remains as an important task. Second, we used only 4 weeks short-term training period. It remains unclear whether or not our developed processing speed training program has benefit using a long-term training period such as 3 or 6 months. To conduct the short- and long-term training periods using the same processing speed training program, we can directly compare benefits of the short- and long- term intervention period. Third, we did not include measurements related to everyday behaviors such as driving. Previous studies demonstrated that cognitive training improves driving skills in elderly people (Edwards et al., 2009b; Nozawa et al., 2015). To generalize the effects of short-term processing speed training, it is necessary to verify the positive effects of the short-term processing speed training on everyday behaviors. Fourth, we used KQTG as the active control group. This method can control the effect of doing a regular cognitive task such as semantic memory or knowledge. However, there is still unclear whether the adaptive approach (the task difficulty was changed based on the performance) or processing speed task in PSTG led to improvements of cognitive performance and emotional state. To specify the key elements of PSTG, in the future study, it should be needed to conduct a RCT using non-adaptive PSTG as an active control group.

## Conclusion

In summary, we newly developed PSTG for use on tablet PCs. We investigated the benefits of a 4-week PSTG on processing speed, inhibition, and depressive mood in healthy elderly people. Our study first provided scientific evidence that PSTG improves processing speed and inhibition performance and reduces depressive mood after the intervention period. Some unresolved issues persist, such as long-term benefits and beneficial effects for everyday behaviors. Our results extended previous findings demonstrating that short-term processing speed training has a small acute effect of processing speed, inhibition and depressive mood in elderly people.

## Ethics Statement

Ethical approval was provided by the Institutional Review Board of the Tohoku University Graduate School of Medicine. This study was conducted according to the principles outlined in the Declaration of Helsinki. Written informed consent was received from each participant.

## Author Contributions

RN designed, developed the study protocol, and calculated the sample size. RN, TS, and HN conducted the study. RN wrote the manuscript with TS, HN, and RK. RK provided advice related to the study protocol. All authors read and approved the final manuscript.

## Conflict of Interest Statement

RK and RN developed the processing speed training game and the knowledge quiz training game with Sharp Corp. Sharp Corp. also provided a tablet PC with training games to participants. After this study, all tablet PCs were returned to Sharp Corp. This study was supported by Sharp Corp. Sources of funding for this study had no involvement in the study design, collection, analysis, interpretation of data, or writing of paper. The other authors declare that the research was conducted in the absence of any commercial or financial relationships that could be construed as a potential conflict of interest.

## Footnotes

1 http://www.consort-statement.org/home/

2 http://www.graphpad.com/quickcalcs/index.cfm
