# Supplementary material for: Small Acute Benefits of 4 Weeks Processing Speed Training Games on Processing Speed and Inhibition Performance and Depressive Mood in the Healthy Elderly People: Evidence from a Randomized Control Trial
Source: Front Aging Neurosci. 2016 Dec 23;8:302. doi: 10.3389/fnagi.2016.00302 (PMC5179514; doi:10.3389/fnagi.2016.00302)
Supplement: Supplementary file 2 [file Table_2.DOCX]

Supplementary Material

**Four weeks processing speed training games improved cognitive functions and emotional states in the healthy elderly people: Evidence from a randomized control trial**

**Rui Nouchi*, Toshiki Saito, Haruka Nouchi, Ryuta Kawashima**

*** Correspondence:** Corresponding Author: rui.nouchi.a4@tohoku.ac.jp

**Supplemental Table 2. Average of maximum levels in the processing speed training game**

|  | Average (Max =8) | SD |
| --- | --- | --- |
| Connection numbers | 7.54 | 1.60 |
| Revere connection numbers | 7.11 | 2.26 |
| Translating figure to number | 7.57 | 1.24 |
| Calculation with whack a mole | 7.77 | 0.97 |
| Lining up numbers | 7.51 | 1.34 |
| Reverse lining up numbers | 6.94 | 2.03 |
| Counting balls | 4.32 | 2.68 |
| Ball-toss game | 4.30 | 3.02 |
| Coping forms | 6.31 | 2.36 |
| Hide and seek | 3.76 | 2.81 |
| Finding shapes | 4.03 | 1.94 |
| Finding letters | 7.54 | 1.60 |
