# Supplementary material for: Small Acute Benefits of 4 Weeks Processing Speed Training Games on Processing Speed and Inhibition Performance and Depressive Mood in the Healthy Elderly People: Evidence from a Randomized Control Trial
Source: Front Aging Neurosci. 2016 Dec 23;8:302. doi: 10.3389/fnagi.2016.00302 (PMC5179514; doi:10.3389/fnagi.2016.00302)
Supplement: Supplementary file 3 [file Table_3.DOCX]

Supplementary Material

**Four weeks processing speed training games improved cognitive functions and emotional states in the healthy elderly people: Evidence from a randomized control trial**

**Rui Nouchi*, Toshiki Saito, Haruka Nouchi, Ryuta Kawashima**

*** Correspondence:** Corresponding Author: rui.nouchi.a4@tohoku.ac.jp

**Supplemental Table 3. Average of maximum stage in the knowledge quiz training game**

| Game name | Average (Max =8) | SD |
| --- | --- | --- |
| Meaning of words | 7.06 | 1.20 |
| Reading of words | 5.88 | 1.85 |
| Kanji idiom | 1.91 | 1.16 |
| Quiz for Japanese literature | 1.53 | 0.51 |
| Quiz for Japanese society | 3.94 | 0.98 |
