# Supplementary material for: Small Acute Benefits of 4 Weeks Processing Speed Training Games on Processing Speed and Inhibition Performance and Depressive Mood in the Healthy Elderly People: Evidence from a Randomized Control Trial
Source: Front Aging Neurosci. 2016 Dec 23;8:302. doi: 10.3389/fnagi.2016.00302 (PMC5179514; doi:10.3389/fnagi.2016.00302)
Supplement: Supplementary file 4 [file Table_4.DOCX]

Supplementary Material

**Four weeks processing speed training games improved cognitive functions and emotional states in the healthy elderly people: Evidence from a randomized control trial**

**Rui Nouchi*, Toshiki Saito, Haruka Nouchi, Ryuta Kawashima**

*** Correspondence:** Corresponding Author: rui.nouchi.a4@tohoku.ac.jp

**Supplemental Table 4. Cognitive function score and mental health score in both groups at post-training**

|  | PSTG group | | KQTG group | |
| --- | --- | --- | --- | --- |
|  | Mean | SD | Mean | SD |
| MMSE (score) | 29.36 | 0.72 | 29.06 | 1.09 |
| Raven (number) | 34.67 | 2.06 | 34.88 | 1.49 |
| LM (score) | 13.14 | 4.32 | 13.68 | 3.68 |
| Cd (number) | 78.75 | 14.30 | 75.00 | 13.59 |
| SS (number) | 38.83 | 7.13 | 37.18 | 5.19 |
| DS-F (score) | 6.14 | 1.17 | 5.88 | 0.98 |
| DS-B (score) | 5.11 | 1.26 | 4.59 | 0.93 |
| LFT (number) | 9.58 | 2.33 | 10.03 | 3.08 |
| CFT (number) | 13.56 | 2.79 | 13.35 | 2.87 |
| rST (number) | 50.67 | 9.77 | 46.85 | 8.60 |
| ST (number) | 36.00 | 8.64 | 31.50 | 8.64 |
| Mental health in SUBI (score) | 39.47 | 5.67 | 38.83 | 6.01 |
| Mental fatigue in SUBI (score) | 52.61 | 5.76 | 52.81 | 5.41 |
| T-A in POMS (score) | 6.47 | 2.48 | 6.42 | 1.83 |
| D in POMS (score) | 4.92 | 2.09 | 5.89 | 2.26 |
| A-H in POMS (score) | 5.47 | 2.16 | 5.69 | 2.12 |
| V in POMS (score) | 6.44 | 3.02 | 6.92 | 2.55 |
| F_I in POMS (score) | 4.11 | 1.75 | 4.11 | 2.09 |
| C in POMS (score) | 2.00 | 1.80 | 2.83 | 2.49 |
| F in POMS (score) | 5.69 | 1.94 | 5.47 | 1.83 |

PSTG = processing speed training game, KQTG = knowledge quiz training game, MMSE = mini mental state examination, Raven = raven’s coloured progressive matrix test, LM = logical memory, Cd =digit symbol coding, SS = symbol search, DS-F = digit span forward, DS-B = digit span backward, LFT = letter fluency task, CFT = category fluency task, rST = reverse stroop test, ST = stroop test, SUBI = subjective well-being inventory, POMS = profile of mood state, T-A = tension-anxiety, D = depression-dejection, A-H = anger-hostility, V = vigor activity, F-I = Fatigue-Inertia, , C = confusion-bewilderment, F = friendliness, SD = standard deviation.
